# Proteomic analysis to identification of hypoxia related markers in spinal tuberculosis: a study based on weighted gene co-expression network analysis and machine learning

**DOI:** 10.1186/s12920-023-01566-z

**Published:** 2023-06-20

**Authors:** Shaofeng Wu, Tuo Liang, Jie Jiang, Jichong Zhu, Tianyou Chen, Chenxing Zhou, Shengsheng Huang, Yuanlin Yao, Hao Guo, Zhen Ye, Liyi Chen, Wuhua Chen, Binguang Fan, Jiahui Qin, Lu Liu, Siling Wu, Fengzhi Ma, Xinli Zhan, Chong Liu

**Affiliations:** The First Affiliated Hospital of Guangxi Medical University, Nanning, China

**Keywords:** Spinal tuberculosis, WGCNA, Machine learning, ssGSEA, Pharmaco-transcriptomic analysis

## Abstract

**Objective:**

This article aims at exploring the role of hypoxia-related genes and immune cells in spinal tuberculosis and tuberculosis involving other organs.

**Methods:**

In this study, label-free quantitative proteomics analysis was performed on the intervertebral discs (fibrous cartilaginous tissues) obtained from five spinal tuberculosis (TB) patients. Key proteins associated with hypoxia were identified using molecular complex detection (MCODE), weighted gene co-expression network analysis(WGCNA), least absolute shrinkage and selection operator (LASSO), and support vector machine recursive feature Elimination (SVM-REF) methods, and their diagnostic and predictive values were assessed. Immune cell correlation analysis was then performed using the Single Sample Gene Set Enrichment Analysis (ssGSEA) method. In addition, a pharmaco-transcriptomic analysis was also performed to identify targets for treatment.

**Results:**

The three genes, namely proteasome 20 S subunit beta 9 (PSMB9), signal transducer and activator of transcription 1 (STAT1), and transporter 1 (TAP1), were identified in the present study. The expression of these genes was found to be particularly high in patients with spinal TB and other extrapulmonary TB, as well as in TB and multidrug-resistant TB (p-value < 0.05). They revealed high diagnostic and predictive values and were closely related to the expression of multiple immune cells (p-value < 0.05). It was inferred that the expression of PSMB9, STAT 1, and TAP1 could be regulated by different medicinal chemicals.

**Conclusion:**

PSMB9, STAT1, and TAP1, might play a key role in the pathogenesis of TB, including spinal TB, and the protein product of the genes can be served as diagnostic markers and potential therapeutic target for TB.

**Supplementary Information:**

The online version contains supplementary material available at 10.1186/s12920-023-01566-z.

## Introduction

According to World Health Organization (WHO) data, *Mycobacterium tuberculosis* infects about 1/4 of the global population, of which approximately 10 million develop active TB and 1.6 million die from it [1–3]. TB is one of the leading causes of death worldwide and poses a serious threat to global public health security [4]. A lung infection caused by *M. tuberculosis* leads to pulmonary TB. Extrapulmonary TB (EPTB) occurs when *M. tuberculosis* infects the spine, lymph nodes, kidney, liver, intestine, joints, brain, and other organs outside the lung [5]. The most common extrapulmonary form of TB is spinal TB, which accounts for half of all bone TB cases [6–8]. Spinal TB could seriously destroy bone and scoliosis and affect neurological function. It has a high refractory, disability, and recurrence rate, which seriously affects the patient’s quality of life [9, 10]. Studies have revealed that patients infected with *M. tuberculosis* develop active TB when their immune system is imbalanced [11], and the incidence rate of EPTB is higher [12]. Granulomas containing large numbers of immune cells, including macrophages, monocytes, T cells, and B cells, form at sites of *M. tuberculosis* infection [13], suggesting that immune cell dysregulation might play a crucial role in TB pathogenesis.

Current research reveals that hypoxia plays a key role in pathological or physiological immune responses. In different immune processes and microenvironments, hypoxia affects inflammation and immunity differently. In pathological conditions, such as chronic inflammation, infection, and tissue ischemia, pathological hypoxia induces dysregulation of immune cells leading to disease progression [14]. In a study, Allison N. Bucşet al. found that Erdman, a strain of *M. tuberculosis*, exhibited greater virulence under hypoxic conditions. Hypoxia may substantially impact bacterial persistence, reactivation, and treatment efficiency [15. A regulatory factor called hypoxia-inducible factor (HIF) plays an essential role in regulating the transcription of immune effector cells. As a result of tissue hypoxia, the HIF pathway is activated [16, 17]. When the body is infected with bacteria, the bacterial oxygen consumption, formation of oxygen-impermeable biofilms, and inflammation-related hypoxia activate HIF and affect the function of immune cells [18–20]. In addition, a study shows that hypoxia can increase the drug resistance of *Pseudomonas aeruginosa* [21].

In this study, We utilized a label-free protein profiling method to analyze the diseased intervertebral disks of patients with spinal TB. We utilized WGCNA and machine learning methods to find key hypoxia-related genes. Besides, various diagnostic and predictive models were constructed to evaluate the diagnostic and predictive values of these key hypoxia-related genes in TB. We also used ssGSEA to identify immune cells associated with spinal tuberculosis and validated the results with data from routine blood tests. In addition, a pharmacotranscriptomic analysis was also performed.

## Materials and methods

### Tissue samples collection

We collected the intervertebral disks from ten patients who underwent spinal surgery at the First Affiliated Hospital of Guangxi Medical University from 2018 to 2020. Five patients with spinal TB were included in the experimental group, and five patients with thoracolumbar disk herniation were included in the control group. There was no evidence of autoimmune diseases, spinal tumors, or other infectious diseases in any of the patients. This study was conducted following the Helsinki Declaration, which passed the ethical review, and obtained informed consent from all patients.

### Label-free quantitative proteomic analysis

The specific steps and processes of the Label-Free Quantitative Proteomic Analysis are as described in our previous research [22], as follows:

#### Sample lysis

The RIPA solution must be prepared right before use and stored in an ice bath to keep it cool. The mixture consists of RIPA lysis buffer, Protease inhibitor cocktail, and 1 mM PMSF (Phenylmethylsulfonyl fluoride). For each 100 mg sample tissue, 1,000 µl of RIPA solution should be thoroughly mixed and homogenized, with sonication at 4 °C for 5 min. Afterwards, centrifugation should be done at 14,000 g for 15 min at 4 °C. The supernatant should then be transferred to a new EP tube and stored in an ice bath.

#### BCA assay

The BCA (Bicin-choninic Acid) Protein Assay Kit instructions indicate that reagent A and reagent B should be mixed at a ratio of 50:1, and added in 160 µl/well to a 96-well plate (with five wells for a calibration curve and one well for a blank). Then 10 µl of each sample (diluted 5–10 times) or calibration standard protein (at five different concentrations) should be added to the respective wells. The plates should be shaken and incubated at 37 °C for 30 min, after which they should be read at 562 nm wavelength. Using the calibration curve, the protein concentration of each sample can be determined.

#### Acetone precipitation

For every sample, 100 µg of protein was taken and diluted to 1 mg/ml in RIPA buffer. Then, 4–6 times the volume of pre-chilled acetone was mixed into the EP tube and shaken in an ice bath for 30 min or left to incubate at -20 °C for the entire night. Following centrifugation at a speed of 10,000 g and 4 °C, the supernatant was carefully discarded, taking care not to disturb the pellet. The sample was then washed twice using 200 µl of cold 80% acetone.

#### Resuspend protein for tryptic digest

Two hundred µl of 1% SDC and 100 mM ABC (ammonium bicarbonate) were added to the EP tube, mixed with a vortex, and spun down. The EP tube was then subjected to sonication for 5 ~ 30 min in a water bath to dissolve the proteins. Five mmol of TCEP (tris 2-carboxyethyl phosphine) was then added to the EP tube and mixed at 55 °C for 10 min. After the sample was cooled down to room temperature (RT), ten mmol of IAA (iodoacetamide) was added in. The EP tube was then incubated in the dark for 15 min. Trypsin (sequence grade) was resuspended in a resuspension buffer to 0.5 µg/µl and incubated at RT for 5 min. A trypsin solution (protein:trypsin = 50:1) was then added to the EP tube. The mixture was well blended and spun down, then incubated at 37 °C with a thermomixer for approximately 8 h or overnight.

#### Cleaning up of SDC

After 2% TFA (Trifluoroacetic Acid, HPLC) was added to the EP tube, SDC was precipitated. After being centrifuged at the highest speed, the supernatant was transferred to a new EP tube. N * 100 µl of 2% TFA was added to the pellet to extract the co-precipitated peptides. This step was repeated twice. The three supernatants were then combined. After being centrifuged at the highest speed for 10–20 min, the supernatant was carefully transferred to a new EP tube, leaving the peptide samples.

#### Peptide desalting for Base-RP fractionation

Buffer A (0.1% FA, H2O, 2% ACN) and Buffer B (0.1% FA, 70% ACN) were prepared. The C18 (3 M) column was then equilibrated using 500 µl of ACN. This was followed by washing it out with 500 µl of 0.1% FA twice. The peptide solution was then added to the column. After low speed centrifugation, liquid (A) was collected. This process was repeated once more, with peptide eluted using 400 µl of 70% ACN and liquid (A) collected. Desalting was performed once again with liquid (A). The two liquids were then combined and dried with a vacuum at either 4 °C or room temperature. Buffer A was then added to re-dissolve the peptide to 1 buffer g/buffer L for LC-MS/MS detection or storage at − 80 °C.

#### Separation via Nano-UPLC and LC-MS/MS

Separate 2 µg peptides from each sample and detect them using nano UPLC coupled with Q-Exactive mass spectrometry. Analyze using a reverse-phase column and a mobile phase composed of solvent A (0.1% FA, 2% ACN) and solvent B (80% ACN, 0.1% FA). Samples are directly loaded onto the chromatographic column by an autosampler and then separated by the column. Analyze peptides for 240 min/sample by LC-MS/MS, using positive ion detection mode with a scanning range of 350–1600 m/z and DDA acquisition method. Use standard parameters for resolution, AGC, maximum IT, NCE, isolation window, and dynamic exclusion time.

#### MaxQuant analysis and LFQ

MaxQuant (1.6.1.0) processed raw MS data using the UNIPROT database. LFQ with trypsin, oxidation [M], and acetyl [protein N-term] modifications were used. Carbamidomethyl [C] was set as the fixed modification (maximum of three variable modifications). Peptides without variable modifications were used for quantification, with an FDR of 0.01. Ten samples were standardized, and missing values were imputed using Perseus software. Protein groups with fewer non-missing values than biological replicates were removed. LFQ quantification results were log-transformed.

### Identification of differentially expressed proteins

To identify differentially expressed proteins between spinal TB and controls, we performed differential analysis of the normalized quantitative results using the “limma” package. | logfc | > 1 and p-value < 0.05 were set as the conditions for screening differentially expressed proteins [23, 24]. To illustrate these differential proteins more clearly, we created a volcano plot and cluster heat map using the “impulse” and “pheatmap” package. All operations were carried out on the R language programming software (version 4.1.1).

### GO/KEGG and DO enrichment analyses

To further explore the biological functions of these differential proteins, we used the “clusterprofiler” package for gene ontology (GO) and Kyoto encyclopedia of genes and genomes (KEGG) enrichment analyses [25–27]. In addition, we also performed a disease ontology (DO) analysis on these differential proteins to reveal the relationship between spinal TB and other diseases [28]. To improve the accuracy of the results, we set the screening conditions as p-value < 0.05 and q-value < 0.05. Finally, the top 10 GO terms, KEGG pathway, and DO terms with the most significant enrichment were visualized.

### Weighted gene co-expression network analysis

Weighted gene co-expression network analysis (WGCNA) is a system biology method used to describe the gene association pattern between different samples. It can be used to identify the gene set with highly synergistic changes and identify the gene set with the strongest correlation with the disease according to the interconnection of the gene set and the association between a gene set and phenotype. It is widely used in the research of diseases and other traits and gene association studies [29]. In this study, we employed the “WGCNA” package to cluster all proteins, automatically select the best soft threshold, and finally obtain each protein module related to the disease.

### Construction of a PPI network of hypoxia-related proteins

In this study, we investigated the role of hypoxia-related proteins in spinal TB by intersecting the two most disease-related modules in WGCNA with a set of all hypoxia-related genes in humans downloaded from the Molecular Signatures Database (version 7.5.1) and differential proteins [29]. Later, the results were used to construct a protein-protein interaction network through the STRING database (version 11.5) and visualized through Cytoscape (version 3.9.0). Finally, a key module in the network was retrieved through the MCODE plugin in Cytoscape software [30].

### Identification of key hypoxia-related proteins and prediction model construction

In order to investigate the transcriptome expression level of hypoxia-related proteins closely related to spinal TB in TB, the GSE144127 dataset, GSE83456 dataset, and GSE147690 dataset related to TB were downloaded from the GEO database (https://www.ncbi.nlm.nih.gov/geo/). The mRNA expression levels of these hypoxia-related proteins in spinal TB and other extrapulmonary TB from the GSE144127 dataset were extracted for differential analysis. Finally, 11 hypoxia-related genes with consistent changes at the transcriptional and protein levels were obtained. We utilized two machine learning methods, LASSO and SVM-REF, to screen these 11 hypoxia-related genes further. LASSO is a regression analysis method that performs variable selection and regularization while fitting a generalized linear model and selects the best variable by the smallestλvalue [31]. This process is achieved through the “glmnet” package. SVM-REF is a powerful feature selection algorithm that continuously eliminates the redundancy between features and finds the optimal feature subset by repeatedly building the model [32]. This process is implemented by the “e1071”, “kernlab” and “caret” packages. Subsequently, we integrated the genes from the LASSO, SVM-REF, and MCODE modules to obtain three important genes. Finally, a diagnostic model was developed using five machine learning techniques, including logistic regression [33], Bayesian logistic regression [34], decision tree [35], random forest [36], and extreme gradient boosting [37], to evaluate the diagnostic value of these three genes in TB disease.

### Immune infiltration analysis

We obtained 28 immune cells and their marker genes from a prior study, used ssGSEA to assess the protein expression matrix through the “GSVA” package, and scored each sample according to the expression of the marker genes to determine the immune cell infiltration level [31]. Finally, using the “limma” and “corrplot” packages, the difference and correlation analyses were performed.

### Blood routine data validation

To further validate the differential analysis of immune cell infiltration findings, we collected lymphocytes, monocytes, and platelets during routine blood examinations from 162 normal patients and 237 patients with spinal TB for statistical analysis. This study adhered to the Declaration of Helsinki guidelines and received approval from the hospital ethics committee.

### Pharmaco-transcriptomic analysis

To provide new solutions for treating multidrug-resistant TB, we conducted a pharmaco-transcriptomic analysis utilizing the DrugBank database (version 5.1.9). DrugBank database integrates the chemical structure and pharmacological action of drugs, as well as the sequence, structure, and physiological pathway of drug action targets [38]. It is an extensive, public web database. Finally, Cytoscape was used to obtain and visualize the effect of drug molecule metabolism on the up- or down-regulation of genes.

### Immunohistochemistry

In this study, 5 cases of intervertebral disc tissue resected during surgery for spinal tuberculosis diagnosed at First Affiliated Clinical Hospital of Guangxi Medical University were taken as a test group, and 5 cases of intervertebral disc tissue resected during surgery for lumbar intervertebral protrusion were taken as the control group. The differences in expression of PSMB9, STAT1, and TAP1 between experimental and control groups were compared by immunohistochemistry. After separating the disc tissue, we immersed it in formalin solution and preserved it within 10 min. We then made immunohistochemical sections and done staining after laboratory operations such as wax sealing, sectioning, antigen repair, antibody hybridization, color development, and tissue sealing. The specimens were observed under the inverted microscope, and the experimental and control group images were collected, respectively. We used Image J software to evaluate the positive rate of all immunohistochemical images and used an independent samples t-test to statistically analyze the positive rate of PSMB9, STAT1, and TAP1 in the experimental group and the control group, respectively, through IBM SPSS Statistics 26.0.

## Results

### Differentially expressed proteins

**Fig. 1:**
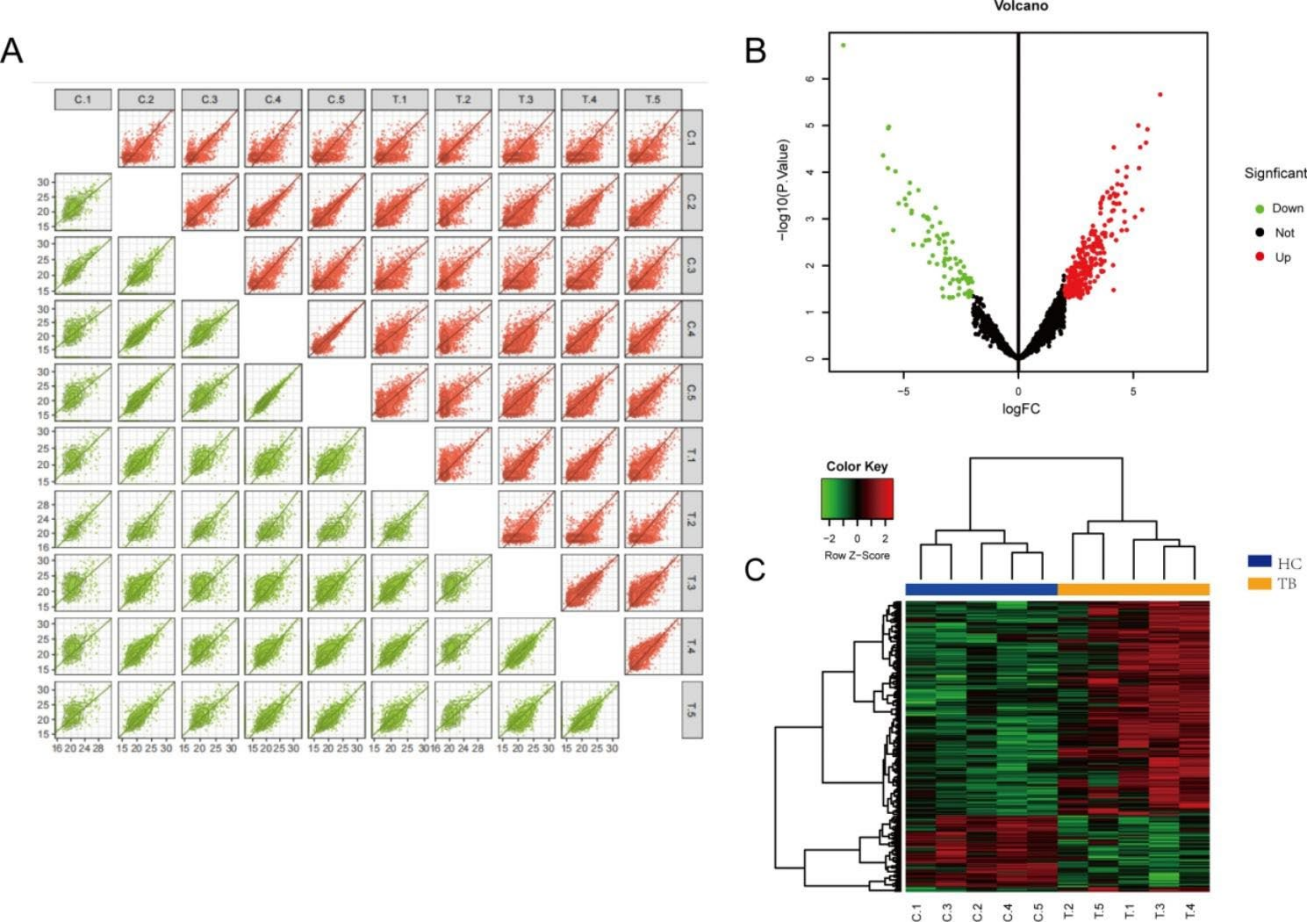
Differentially expressed proteins. (**A**) The quantitative repeatability analysis between samples. (**B**) The cluster heat map of differentially expressed proteins. (**C**) The volcano plot of differentially expressed proteins

Following label-free quantitative proteomic analysis, we obtained 1965 quantifiable proteins. The quantitative repeatability analysis between samples revealed that the quantitative experiment had good sensitivity and reliability (Fig. 1A). According to the screening conditions, we obtained 350 differentially expressed proteins, which could be clearly distinguished by volcano plot (Fig. 1B) and cluster heat map (Fig. 1C). Furthermore, the cluster heat map also indicated that these differential proteins could distinguish well between the spinal TB and control groups.

### GO/KEGG and DO enrichment analyses

**Fig. 2:**
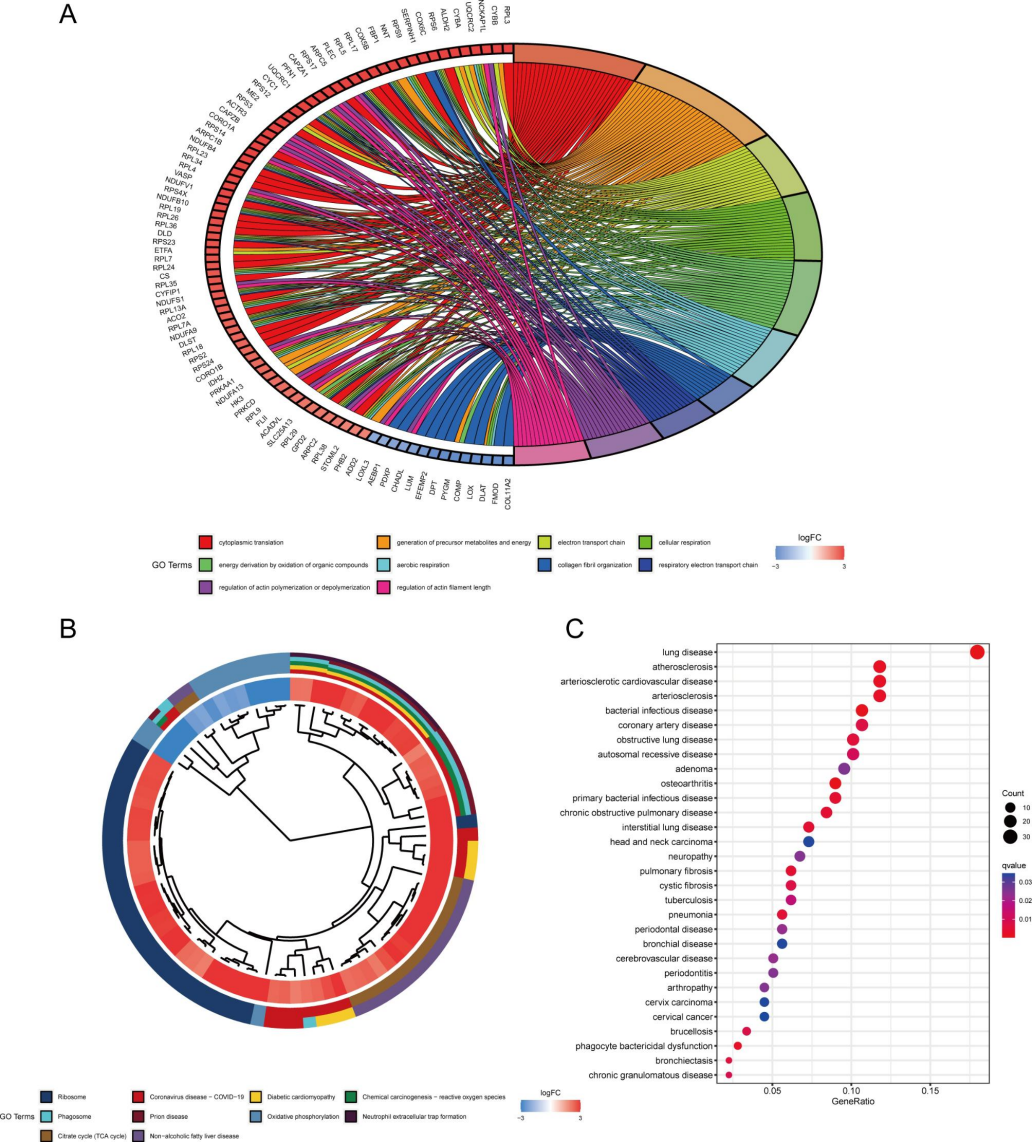
GO/KEGG and DO enrichment analyses. (**A**) The top 10 entries of GO enrichment analysis for differentially expressed proteins. (**B**) The top 10 entries of the KEGG pathway enriched by the differentially expressed proteins. (**C**) The top 30 entries of DO analysis enriched by the differentially expressed proteins

Through GO enrichment analysis, we found that these differentially expressed proteins are primarily involved in cytoplasmic translation, generation of precursor metabolites and energy, electron transport chain, cellular respiration, oxidation of organic compounds to produce energy, aerobic respiration, collagen fibril organization, and other processes (Fig. 2A). KEGG pathway analysis showed that these differentially expressed proteins were primarily related to a ribosome, coronavirus disease (COVID-19), chemical carcinogenesis-reactive oxygen species, phagosome, oxidative phosphorylation, neutrophil extracellular trap formation, citrate cycle (TCA cycle) and other pathways (Fig. 2B). DO analysis found that these differentially expressed proteins were not only linked to pulmonary disease but also linked to osteoarthritis, bacterial infectious disease, atherosclerosis, arteriosclerotic cardiovascular disease, phagocyte bactericidal dysfunction, and other diseases. This provides novel insights into the etiology and comorbidities of spinal TB (Fig. 2C).

### WGCNA and identification of key modules

**Fig. 3:**
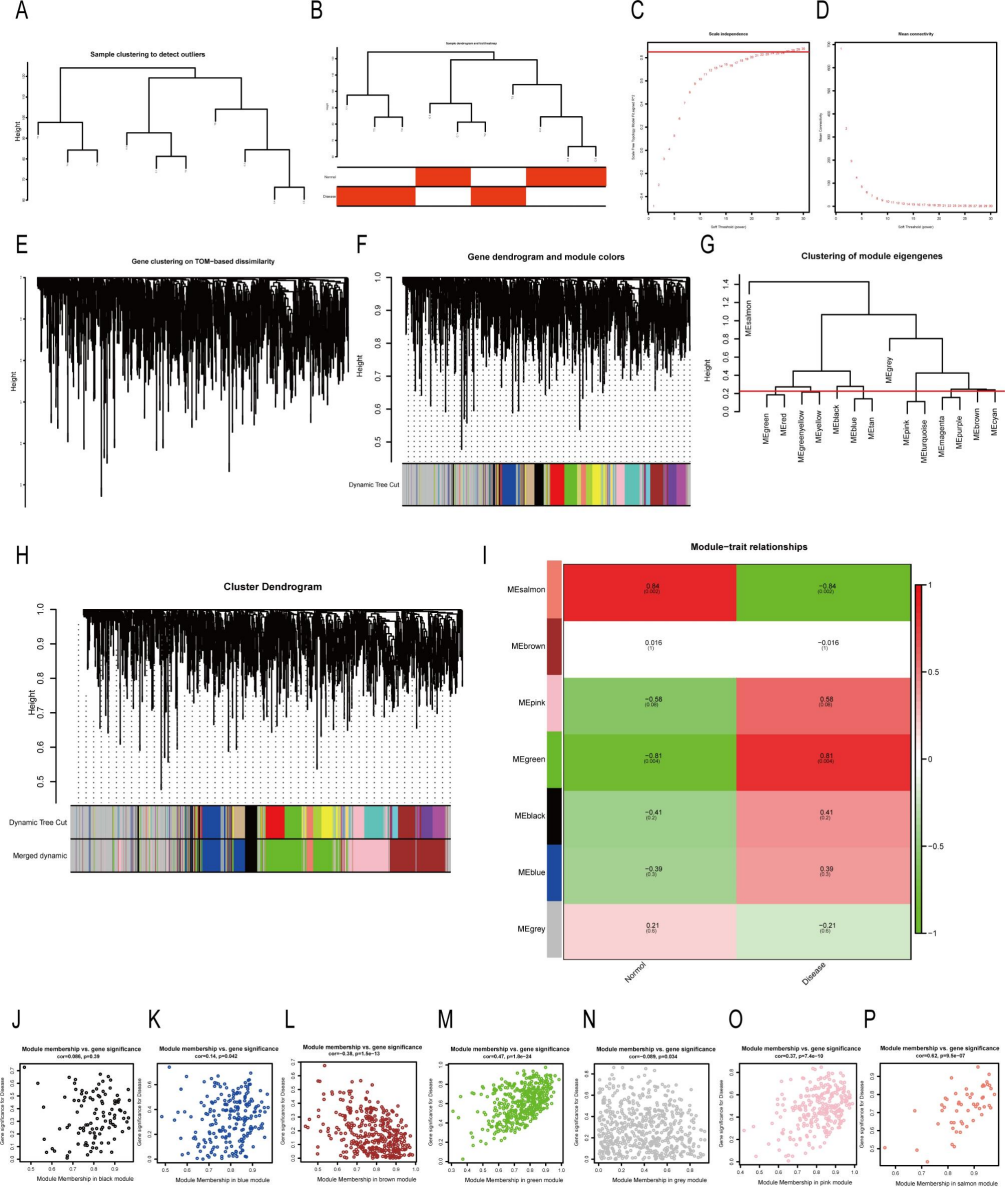
Results of weighted gene co-expression network analysis. (**A-P**) The entire WGCNA process, from sample clustering to correlation analysis, looking for the genes in the modules most associated with the disease

WGCNA could cluster genes with similar expression patterns, analyze the correlation between modules and specific traits or phenotypes, and identify the molecular markers that are strongly correlated with diseases. It is an advanced method frequently employed for bioinformatics analysis. Following analysis, we found that the two modules, “salmon” and “green,“ were highly correlated with spinal TB (Fig. 3A-I), and the gene expression in most modules also showed a significant correlation (Fig. 3J-P).

### PPI network of hypoxia-related proteins

**Fig. 4:**
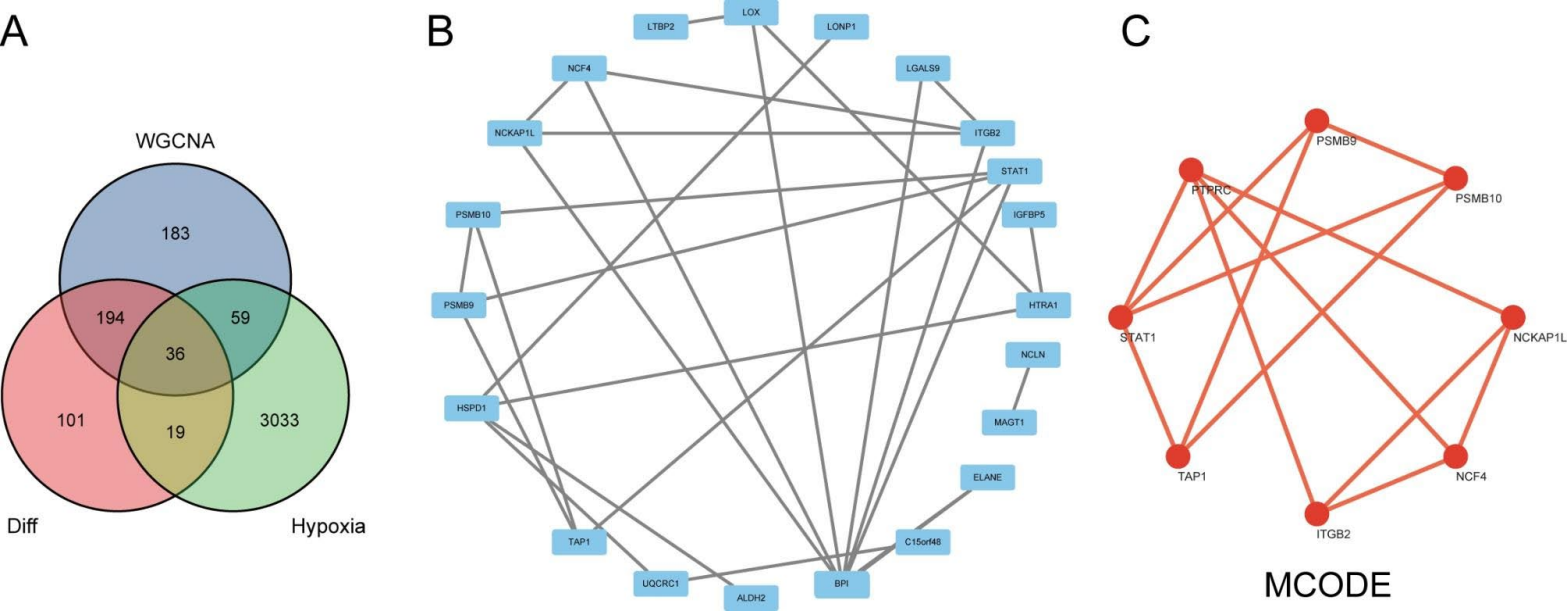
The PPI network of hypoxia-related proteins. (**A**) The results of taking the intersection of the differentially expressed proteins, the hypoxia-associated genes, and the genes from the two modules most associated with the disease. (**B**) A protein-protein interaction network of hypoxia-related proteins. (**C**) The key module in the network

We intersected the proteins in the two modules of “salmon” and “green” in WGCNA with 3147 hypoxia-related genes and 350 differential proteins screened by our study. Finally, 36 hypoxia-related proteins were obtained in total (Fig. 4A). We constructed a protein-protein interaction network using the string database with 22 points and 27 edges (Fig. 4B). Through the MCODE plugin, we found that there is only one key module in the network (Fig. 4C).

### The key hypoxia-related proteins and prediction models

**Fig. 5:**
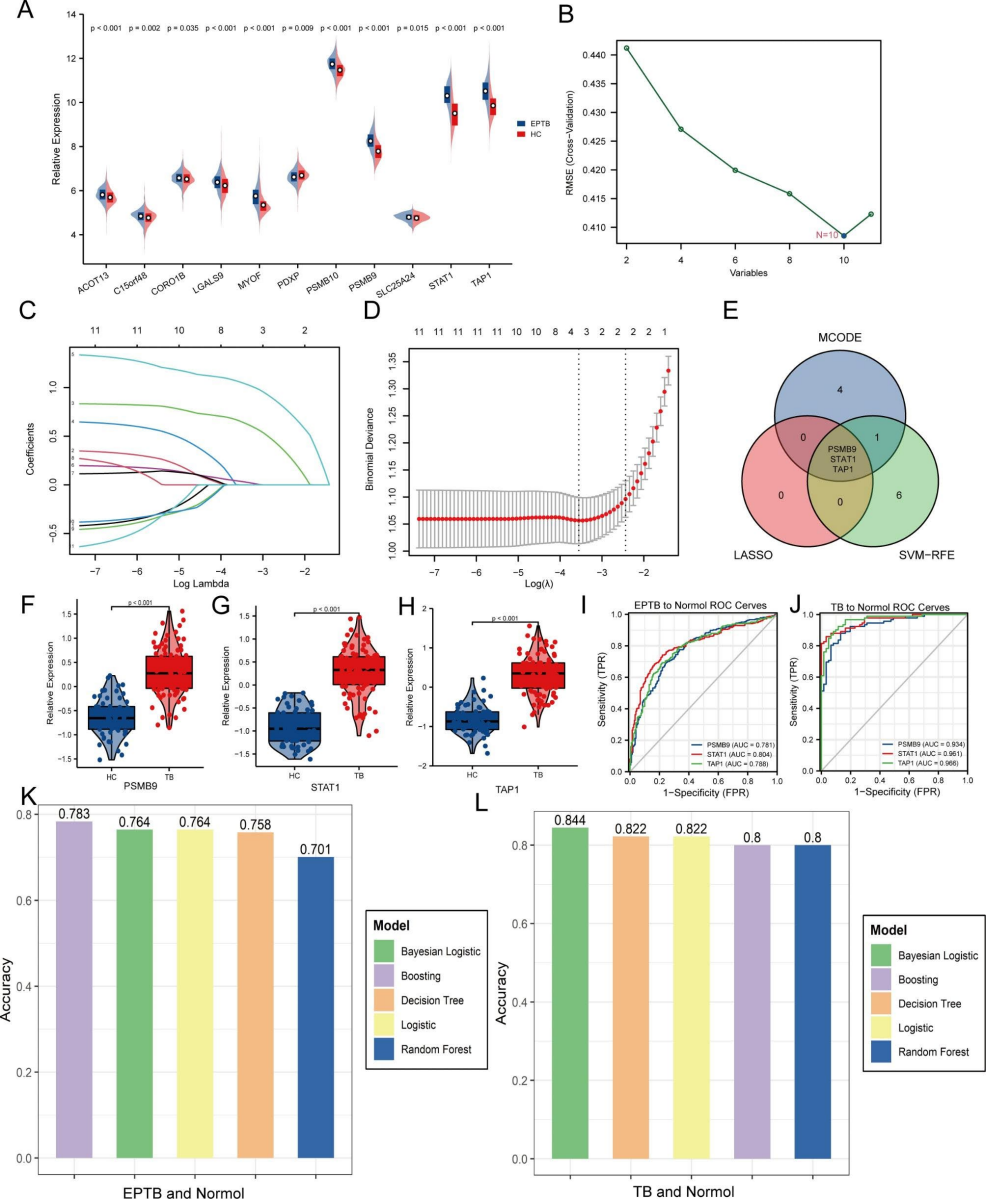
The key hypoxia-related proteins and prediction models. (**A**) Differential expression of 11 genes in extrapulmonary TB and control group in the GSE144127 dataset. (**B**) SVM-REF algorithm for screening key genes. (**C**) LASSO coefficient spectrum of 11 differentially expressed genes selected by optimal. (**D**) Selection of the best parameter. (**E**) PSMB9, STAT1, and TAP1 were screened by two algorithms and MCODE. (**F-H**) Differential expression of PSMB9, STAT1, and TAP1 between TB group and control group in the GSE83456 dataset. (**I, J**) Diagnostic ROC curves of PSMB9, STAT1, and TAP1 in extrapulmonary TB and TB. (**K, L**) Accuracy of PSMB9, STAT1, and TAP1 prediction models based on 5 machine learning algorithms for extrapulmonary TB and TB.

To further explore the role of hypoxia-related genes in TB, we analyzed the GSE144127 datasets. We found that the transcriptional levels of 11 genes in these 36 hypoxia-related genes were consistent with the protein expression levels (10 up-regulation and 1 down-regulation). The difference in transcriptional level was significant, in extrapulmonary TB and the control group (Fig. 5A). These 11 genes were further screened in extrapulmonary TB and control groups using LASSO and SVM-REF machine learning (Fig. 5B-D) and then intersected with the key modules extracted by the MCODE plugin. Finally, three genes, PSMB9, STAT1, and TAP1, were obtained (Fig. 5E). The GSE83456 dataset revealed significant differences in these three genes between the TB and control groups (Fig. 5F-H). In addition, in the GSE144127 dataset, the AUC of PSMB9, STAT1, and TAP1 in extrapulmonary TB and the control group were 0.781, 0.804, and 0.788 (Fig. 5I). In the GSE83456 dataset, the AUC of PSMB9, STAT1, and TAP1 genes in the TB and control groups were as high as 0.934, 0.961, and 0.966 (Fig. 5J). All these three genes have high diagnostic value for TB and may play a crucial role in the pathogenesis of TB.

Finally, the five machine learning methods of logistic regression, Bayesian logistic regression, decision tree, random forest, and extreme gradient boosting were used to build a prediction model based on these three genes. In the GSE144127 dataset, the accuracies in extrapulmonary TB and the control group were 0.764, 0.764, 0.758, 0.701 and 0.783, respectively (Fig. 5K). In the GSE83456 dataset, the accuracies of pulmonary TB and the control group were 0.822, 0.844, 0.822, 0.8, and 0.8 (Fig. 5L). Comparatively, we can observe that the machine learning method extreme gradient boosting has the highest prediction accuracy for extrapulmonary TB, which is 0.783, and Bayesian logistic regression has the highest prediction accuracy for pulmonary TB, which is 0.844.

### Immune infiltration analysis

**Fig. 6:**
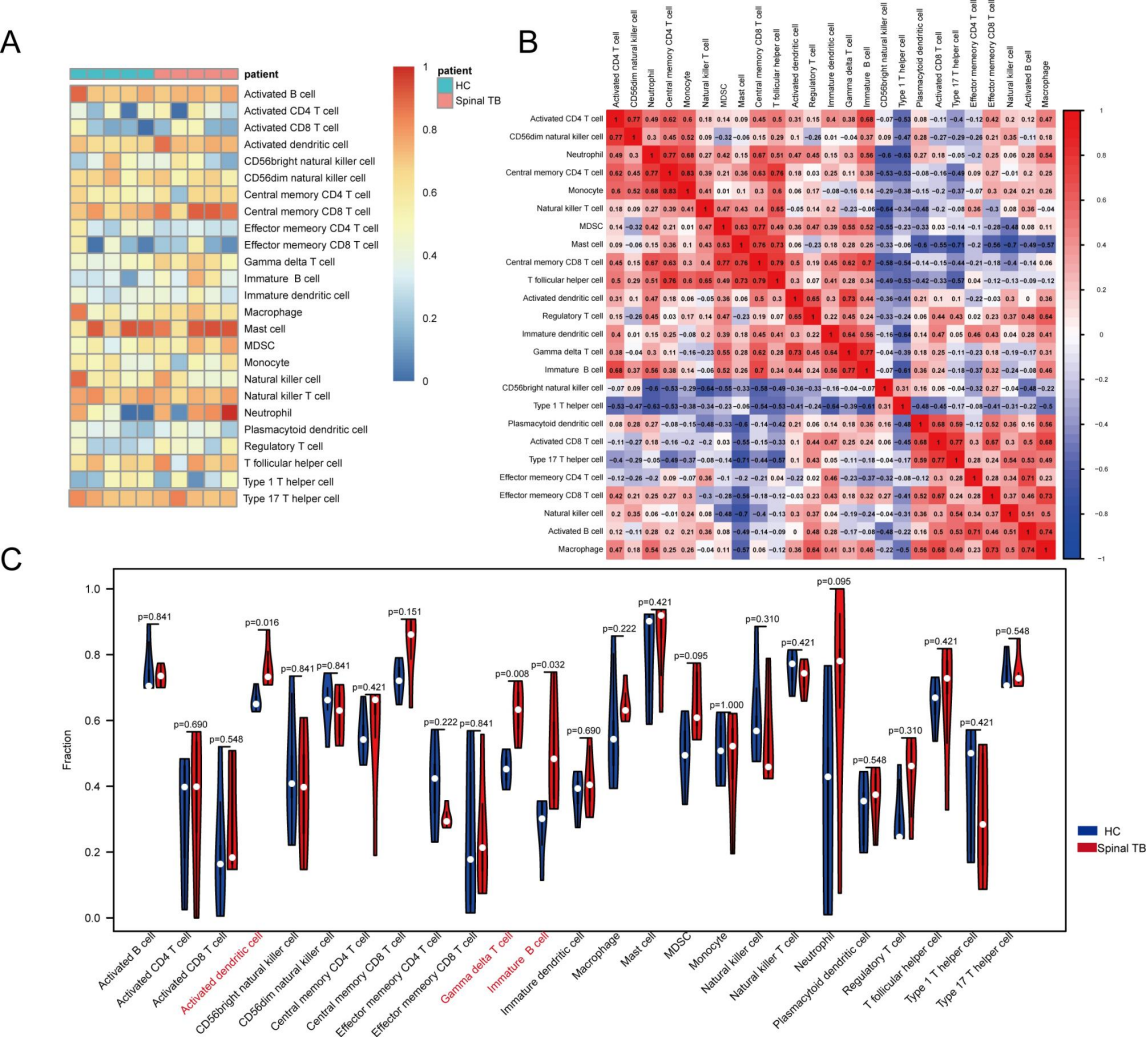
Immune infiltration analysis. (**A**) Heat map of the landscape of 25 immune cell subpopulations infiltration. (**B**) Heat map of correlation between immune cells. (**C**) Violin plot of immune cell differences between disease group and control group

By ssGSEA analysis, we obtained 25 types of infiltrating immune cells in all protein samples (Fig. 6A). Through the correlation heat map, we can observe that activated dendritic cells with gamma delta T cells possess a strong positive correlation, r = 0.73, and gamma delta T cells with immature B cells also possess a strong positive correlation, r = 0.77. Monocytes and most lymphocytes also have a more significant correlation (Fig. 6B). Differential analysis showed that most immune cells were highly infiltrated in the disease group, and the activated dendritic cells, gamma delta T cells, and immaturity B cells were significantly different between the spinal TB group and control group (p-value < 0.05) (Fig. 6C).

### Correlation of hypoxia-related genes PSMB9, STAT1, and TAP1 with immune cells

**Fig. 7:**
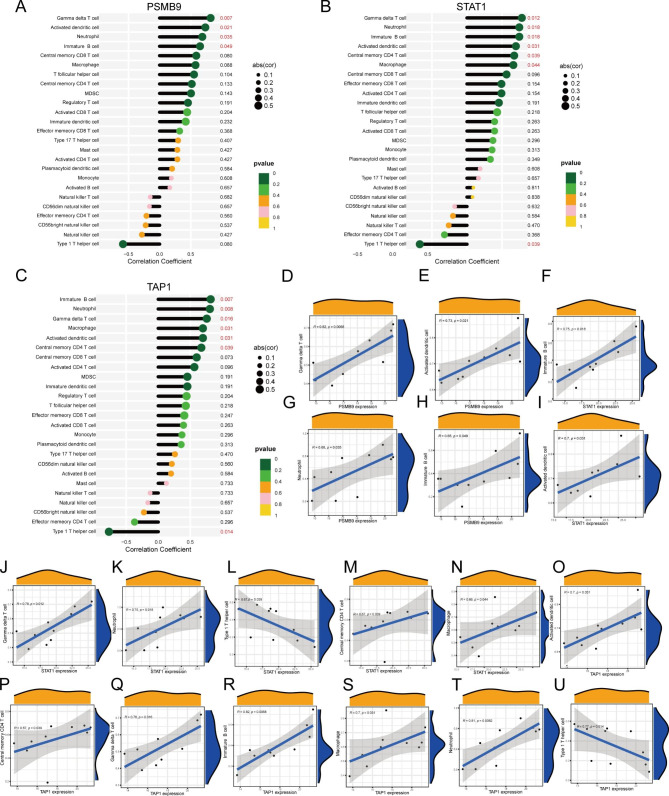
Correlation of PSMB9, STAT1, and TAP1 with immune cells. (**A-C**) Lollipop plot of correlation of PSMB99, STAT1, and TAP1 with immune cells. (**D-U**) Scatter plot of significant correlation between PSMB99, STAT1, and TAP1 with immune cells

Following correlation analysis (Fig. 7), we found that PSMB9, STAT1, and TAP1 significantly correlated with activated dendritic cells, gamma delta T cells, immature B cells, and neutrophils. In addition, STAT1 and TAP1 were also significantly positively correlated with central memory CD4 T cells and macrophages while negatively correlated with Type 1 T helper cells. PSMB9 and STAT1 had the strongest and most significant correlation with gamma delta T cells, while TAP1 had the strongest and most significant correlation with immature B cells. This suggests that these key genes and immune cells might play an important role in the pathogenesis of TB, including spinal TB (Fig. 7A-U).

### Blood routine data validation

**Fig. 8:**
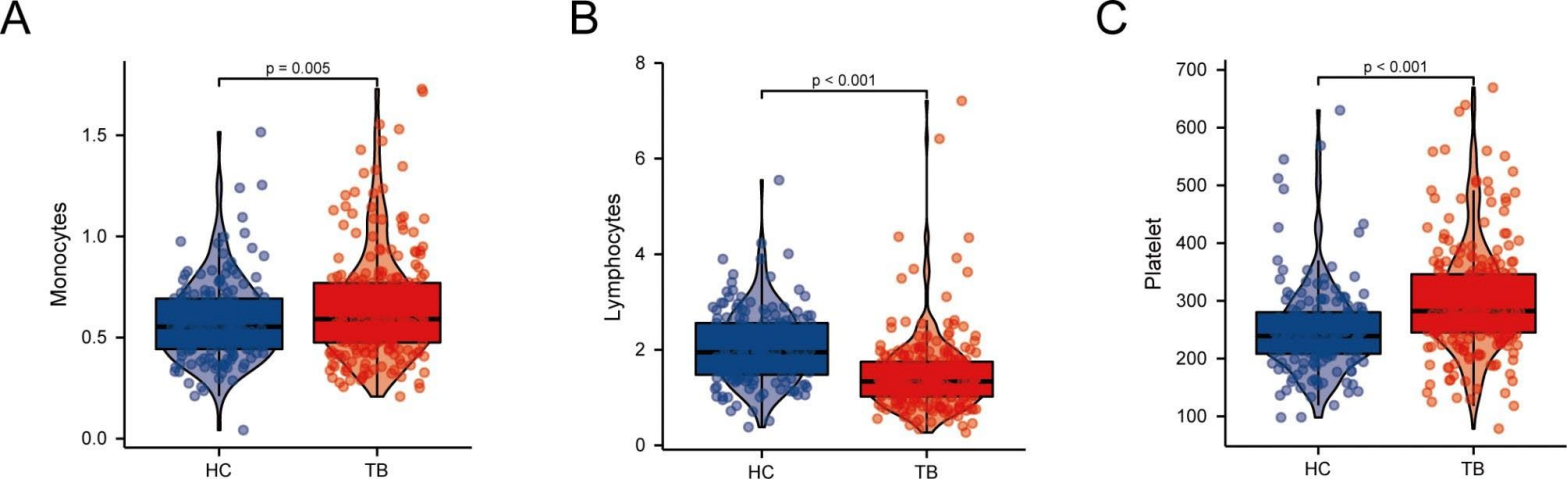
Routine blood tests. (**A-C**) The results of routine blood tests for monocytes, platelets, and lymphocytes in 162 normal patients and 237 patients with spinal TB.

Through a statistical analysis of the blood routine examination of 162 normal patients and 237 patients with spinal TB, we found that the monocytes and platelets in the spinal TB group were higher in comparison to the normal control group. In contrast, the lymphocytes in the normal control group were higher in comparison to the spinal TB group, and the difference was statistically significant (p-value < 0.05) (Fig. 8A-C). According to our immune cell infiltration results, based on ssGSEA analysis, the monocytes and macrophages had higher infiltration levels in the disease group. This finding was proven to be accurate through routine blood data.

### Pharmaco-transcriptomic analysis

**Fig. 9:**
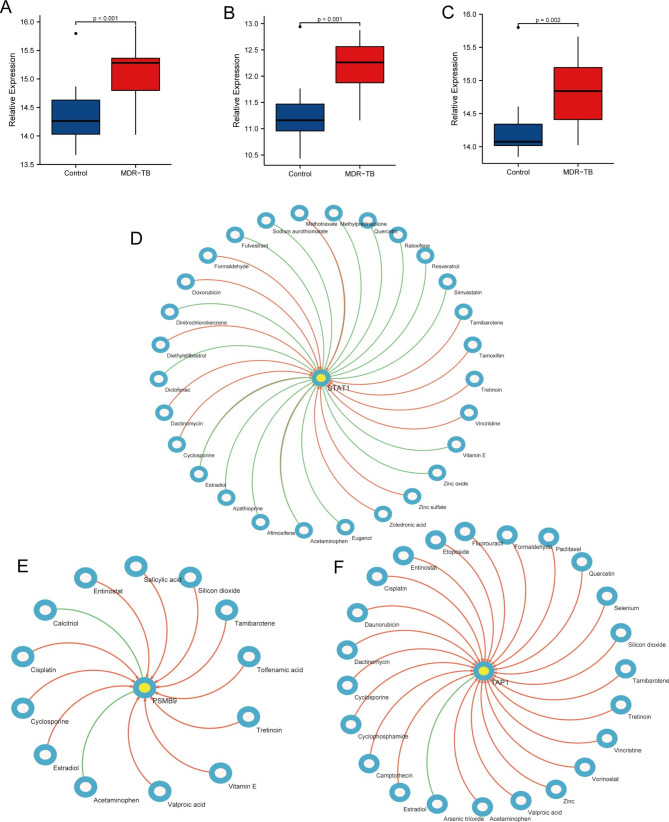
Pharmaco-transcriptomic analysis. (**A-C**) Differential expression of PSMB9, STAT1, and TAP1 between the multidrug-resistant TB group and the control group. (**D-F**) The pharmaco-transcriptomic analysis of PSMB9, STAT1, and TAP1.

In the GSE147690 dataset, we found that PSMB9, STAT1, and TAP1 were also highly expressed in the multidrug-resistant TB group, and the difference was very statistically significant (p-value < 0.01) (Fig. 9A-C). PSMB9, STAT1, and TAP1 may be potential therapeutic targets for multidrug-resistant TB. Therefore, we performed pharmaco-transcriptomic analysis and found that 11 drug compounds, such as estradiol, cyclosporine, and cisplatin, can upregulate the expression of PSMB9. At the same time, acetaminophen and calcitriol can down-regulate the expression of PSMB9. Cyclosporine, dactinomycin, diethylstilbestrol, and other 11 drug compounds can upregulate the expression of STAT1. In contrast, afimoxifene, azathioprine, diclofenac, and other 14 kinds of drug compounds can down-regulate the expression of STAT1, and acetaminophen, estradiol, and methotrexate have effects on the up- and downregulation of STAT1. Dactinomycin, daunorubicin, camptothecin, and other 22 drug compounds can upregulate the expression of TAP1, while arsenic trioxide can downregulate the expression of TAP1 (Fig. 9D-F). This will help us in providing new insights into the treatment of multidrug-resistant TB.

### Immunohistochemical analysis results

**Fig. 10:**
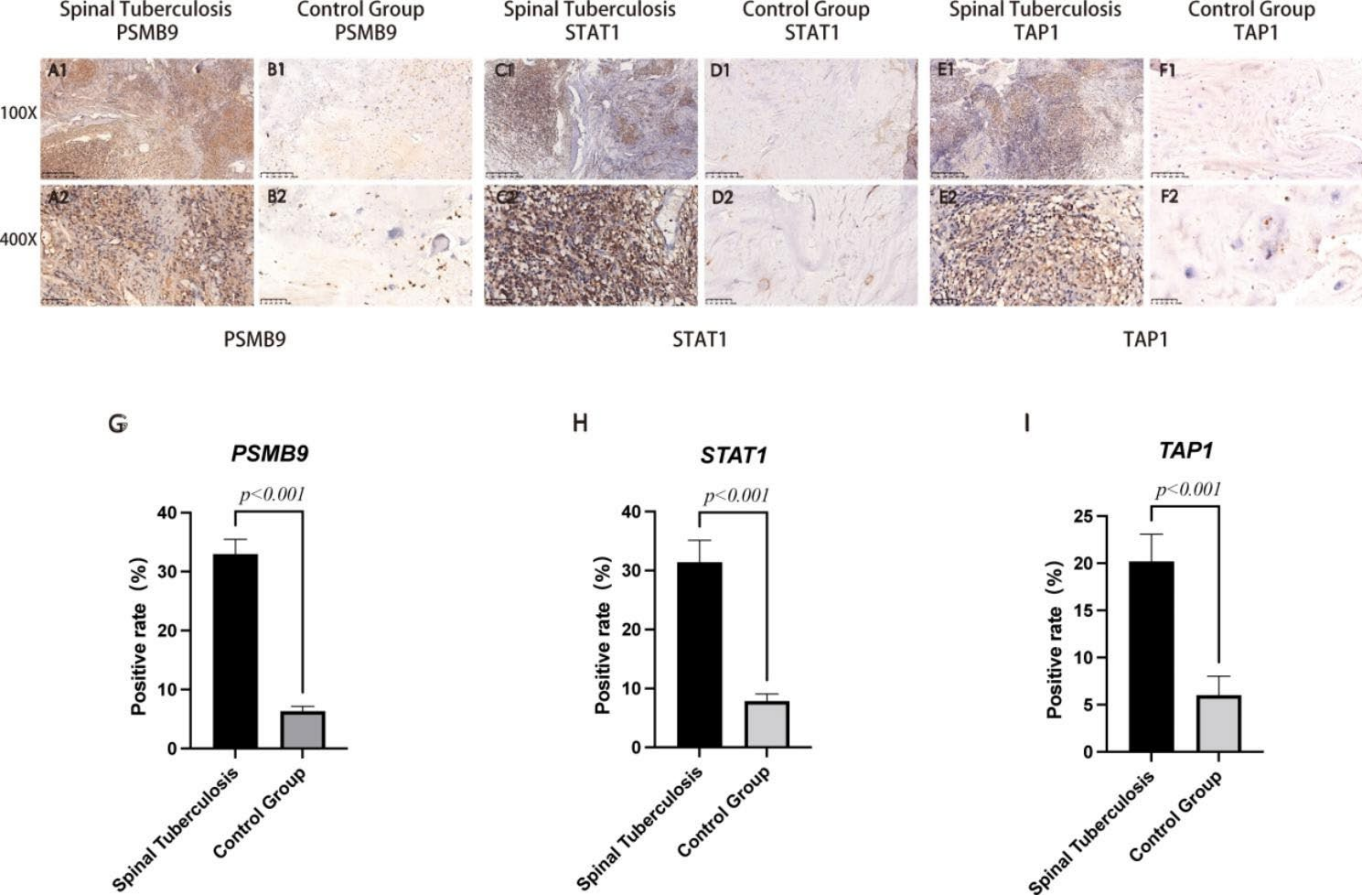
Immunohistochemical staining analysis. (**A-F**) Shows the specific expression of PSMB9, STAT1, and TAP1 in spinal TB group and the control group. (**G-I**) Shows the statistical analysis results of the positivity rate between spinal TB group and the control group.

Immunohistochemical staining of PSMB9, STAT1, and TAP1 was performed in 5 patients with spinal tuberculosis and 5 patients with lumbar disc herniation. The results showed that the specific expressions of PSMB9, STAT1, and TAP1 in the experimental group were significantly higher than in the control group (Fig. 10A-F). We used Image J software to detect the positive rate of immunohistochemical images. The positive rate data of PSMB9, STAT1, and TAP1 were imported into SPSS 26.0, and the difference between the two groups was statistically analyzed by independent sample t-test. The positive rates of PSMB9, STAT1, and TAP1 genes in the experimental group were significantly higher than those in the control group (p-value < 0.001) (Fig. 10G-I). It showed that PSMB9, STAT1, and TAP1 were differentially expressed in the experimental and control groups. This result confirms the accuracy of our analysis.

## Discussion

Granuloma is an important feature of TB, and it is also a place where *M. tuberculosis* obtains nutrients and evades immunity, and plays a key role in the spread of TB infection [39, 40]. Studies suggest that *M. tuberculosis* granulomas may be in a hypoxic environment in which *M. tuberculosis* enters a non-replicating “quiescent” state, thereby enhancing bacterial resistance to antibiotics [41]. Hua Yang et al. found that *M. tuberculosis* can secrete fatty acid-degrading protein A under hypoxic conditions, regulate fatty acid metabolism, and inhibit the secretion of pro-inflammatory cytokines, thereby inhibiting host immunity so that *M. tuberculosis* could survive in the granuloma and persist in the host infection [42]. Therefore, the molecular mechanism of hypoxia-related genes in tuberculosis infection deserves further exploration.

By analyzing the differentially expressed proteins between the spinal TB group and the control group, we found that in the GO enrichment analysis, these differential proteins were mainly concentrated in the generation of precursor metabolites and energy, cellular respiration, oxidation of organic compounds to produce energy, aerobic respiration, respiratory electron transport chain, and reactive oxygen species metabolic process. KEGG pathway analysis also showed that these differential proteins mainly concentrated in the ribosome, chemical carcinogenesis-reactive oxygen species, oxidative phosphorylation, and citrate (TCA cycle). Ribosomal stability is very important for the persistence and latent infection of mycobacteria. Under hypoxic conditions, ribosome-associated factor during hypoxia (Rafh) is the primary factor leading to the hypoxic survival of mycobacteria mediated by response regulator dose [43]. All these results indicate that hypoxia is closely related to the pathogenesis of TB.

In this study, we screened out three key hypoxia-related genes, PSMB9, STAT1, and TAP1, which were highly expressed at the protein and transcriptional levels in spinal TB. Notably, previous studies have shown that PSMB9, STAT1, and TAP1 are all associated with TB. A meta-analysis integrating the transcriptional expression dataset of whole blood of multiple hosts and integrating and comparing different data through the network method found that there is a highly active core gene group in TB, which is composed of 380 genes, of which STAT1 and PSMB9 are the key hubs of the gene group [44]. PSMB9 is an immunoproteasome subunit involved in MHC class I antigen presentation, and the expression of this gene is induced by inflammatory factors, such as interferon-gamma [45, 46]. Tetsuaki Shoji et al. found that in cisplatin-resistant lung cancer cell line models, the transcription levels of PSMB8 and PSMB9 were highly expressed, and the protein expression levels were also significantly increased. After treatment with immunoproteasome inhibitors, it was found that immunoproteasomes may be an effective therapeutic target for some cisplatin-resistant lung cancers [47]. STAT1 is a signal transducer and activator of transcription 1, a member of the STAT protein family [48]. This protein can be activated by ligands, such as interferon-alpha and interferon-gamma, and plays an important role in the immune response to viral, fungal, and mycobacterial pathogens [49, 50]. STAT1 transcriptional up-regulation in severe COVID-19 patients is a potential predictive biomarker and target for certain interferon pathway-targeted therapies [51]. Tuo Liang et al. also found that STAT1 is related to the pathogenesis of spinal TB and other extrapulmonary TB, which may be involved in M1-macrophage polarization and then cause bone destruction. It is an important biomarker of tuberculosis and a potential therapeutic target [52]. The full name of TAP1 is transporter 1, an ATP binding cassette subfamily B member. In the process of antigen processing and presentation, heterodimer transporters related to antigen processing (TAP) transport peptides produced by immunoproteasome to the endoplasmic reticulum to play immune function. TAP1 and PSMB9 are involved in the formation of heterodimer transporters and immune proteasomes, respectively. When TAP is dysfunctional, pathogenic microorganisms can escape immune surveillance [53, 54]. Several studies have shown that abnormalities in the TAP1 gene are closely associated with pulmonary TB [55, 56]. In this study, PSMB9, STAT1, and TAP1 have high diagnostic and predictive values for both extrapulmonary TB and TB. These results indicate that PSMB9, STAT1, and TAP1 may play a role in the pathogenesis of TB, such as spinal TB.

In addition, PSMB9, STAT1, and TAP1 were also significantly upregulated in the multidrug-resistant TB group. Pharmaco-transcriptomic analysis showed that estradiol, cyclosporine, cisplatin, and other drug compounds could upregulate the expression of PSMB9, while acetaminophen and calcitriol can down-regulate PSMB9 expression. Cyclosporine, dactinomycin, diethylstilbestrol, and other drug compounds can upregulate the expression of STAT1, while 14 kinds of drug compounds, such as afimoxifene, azathioprine, and diclofenac can down-regulate the expression of STAT1, and acetaminophen, estradiol, and methotrexate have effects on the up and down regulation of STAT1. Dactinomycin, daunorubicin, camptothecin, and other drug compounds can upregulate the expression of TAP1, while arsenic trioxide can down-regulate the expression of TAP1. Cyclosporine is an important immunosuppressant. Its main mechanism is to inhibit the activity of the immune system by inhibiting the activity and growth of T cells [57]. Delayed activation of T lymphocytes and insufficient secretion of related cytokines can lead to pathogenic inflammation, increased bacterial load, spread of infection, and severe disease progression [58, 59]. Therefore, T lymphocytes play an important role in immune protection against Mb infection. Many studies have also shown that cyclosporin is associated with an increased risk of activation of TB and latent tuberculosis infection [60]. In this study, we found that cyclosporin can upregulate the expression of PSMB9 and STAT1, which may be one of the mechanisms of cyclosporin-induced increased risk of activation of tuberculosis disease. Calcitriol is the “active metabolite” of vitamin D3. An in vitro study showed that it has antibacterial properties and inhibits the production of pro-inflammatory cytokines [61]. In addition, calcitriol also plays a role in host defense against mycobacterium tuberculosis infection by inducing autophagy of antimicrobial peptides (AMP) and/or colonized macrophages [62]. Klauer et al. first proved that calcitriol could inhibit pathogenic Mycobacterium tuberculosis proliferation in human macrophages [63]. This provides a new reference for the treatment of multidrug-resistant TB.

TB is closely related to the immune response in the body but the immune mechanism of anti-*M. tuberculosis* antibodies are not completely clear [64]. By analyzing the ssGSEA data, we described the immune cell infiltration of spinal TB. We found that activated dendritic cells, gamma delta T cells, and immature B cells were different in the spinal TB group and the control group, and they were significantly positively correlated with PSMB9, STAT1, and TAP1. Dendritic cells have the function of activating and stabilizing T lymphocytes and B lymphocytes and can differentiate into different immune cells, participate in cellular and humoral responses, and also form complexes with multifunctional APCs, which play a key role in antipathogen activity; they are one of the most important immune regulatory cells [65, 66]. Dendritic cells play a role in granuloma formation by inducing the migration of natural killer (NK) cells and T cells in vitro under the stimulation of *M. tuberculosis* [67]. Gamma delta T cells are unconventional T cells that play an important role in recognizing foreign pathogens and stress signals of infected cells [68–70]. In tuberculosis, γδT cells can rapidly recognize *M. tuberculosis* antigens, respond to the BCG vaccine, inhibit the growth of mycobacteria, and are potential vaccine targets against TB [71]. We also found a significant positive correlation between STAT1 and macrophage, which once again demonstrated that STAT1 might induce M1-macrophage polarization to cause bone destruction in spinal TB. In addition, we analyzed the differences of monocytes/macrophages in patients with spinal TB through the blood routine examination data of 162 normal patients and 237 patients with spinal TB and found that the number of monocytes/macrophages in the disease group was significantly higher than that of normal control groups. This observation verifies the obtained results.

Similar to other studies, our study also had limitations. First, the sample size was inadequate. Taking into account the analysis of large samples, we only used five pairs of 10 samples for the protein park test, which was insufficient. Second, there are limitations in using routine blood data to check differential immunocyte analysis; tissue-based flow cytometry should be used for further verification. In addition, we do not have more laboratory analysis to verify our results, and this study should be further verified through cell and animal experiments.

## Conclusion

PSMB9, STAT1, and TAP1, might play a key role in the pathogenesis of TB, including spinal TB, and the protein product of the genes can be served as diagnostic markers and potential therapeutic target for TB.

## Electronic supplementary material

Below is the link to the electronic supplementary material.

Supplementary Material 1

Supplementary Material 2

Supplementary Material 3

## Data Availability

The original contributions presented in the study are included in the article/supplementary material. Further inquiries can be directed to the corresponding author.

## References

[1] Pai M, Behr MA, Dowdy D, Dheda K, Divangahi M, Boehme CC (2016). Tuberculosis Nat Rev Dis Primers.

[2] Suarez I, Funger SM, Kroger S, Rademacher J, Fatkenheuer G, Rybniker J (2019). The diagnosis and treatment of tuberculosis. Dtsch Arztebl Int.

[3] Furin J, Cox H, Pai M, Tuberculosis (2019). Lancet.

[4] Sharma A, Machado E, Lima KVB, Suffys PN, Conceicao EC (2022). Tuberculosis drug resistance profiling based on machine learning: a literature review. Braz J Infect Dis.

[5] Sharma SK, Mohan A (2004). Extrapulmonary tuberculosis. Indian J Med Res.

[6] Weng CY, Ho CM, Dou HY, Ho MW, Lin HS, Chang HL (2013). Molecular typing of Mycobacterium tuberculosis isolated from adult patients with tubercular spondylitis. J Microbiol Immunol Infect.

[7] Gorse GJ, Pais MJ, Kusske JA, Cesario TC (1983). Tuberculous spondylitis. A report of six cases and a review of the literature. Med (Baltim).

[8] Nussbaum ES, Rockswold GL, Bergman TA, Erickson DL, Seljeskog EL (1995). Spinal tuberculosis: a diagnostic and management challenge. J Neurosurg.

[9] Garcia-Rodriguez JF, Alvarez-Diaz H, Lorenzo-Garcia MV, Marino-Callejo A, Fernandez-Rial A, Sesma-Sanchez P (2011). Extrapulmonary tuberculosis: epidemiology and risk factors. Enferm Infecc Microbiol Clin.

[10] Jain AK (2010). Tuberculosis of the spine: a fresh look at an old disease. J Bone Joint Surg Br.

[11] Cliff JM, Kaufmann SH, McShane H, van Helden P, O’Garra A (2015). The human immune response to tuberculosis and its treatment: a view from the blood. Immunol Rev.

[12] Tahseen S, Khanzada FM, Baloch AQ, Abbas Q, Bhutto MM, Alizai AW (2020). Extrapulmonary tuberculosis in Pakistan- A nation-wide multicenter retrospective study. PLoS ONE.

[13] Cronan MR, Matty MA, Rosenberg AF, Blanc L, Pyle CJ, Espenschied ST (2018). An explant technique for high-resolution imaging and manipulation of mycobacterial granulomas. Nat Methods.

[14] Taylor CT, Colgan SP (2017). Regulation of immunity and inflammation by hypoxia in immunological niches. Nat Rev Immunol.

[15] Bucsan AN, Veatch A, Singh DK, Akter S, Golden NA, Kirkpatrick M et al. Response to Hypoxia and the Ensuing Dysregulation of inflammation impacts Mycobacterium tuberculosis Pathogenicity. Am J Respir Crit Care Med. 2022. 10.1164/rccm.202112-2747OC PMC9718519 35412961

[16] Taylor CT, Doherty G, Fallon PG, Cummins EP (2016). Hypoxia-dependent regulation of inflammatory pathways in immune cells. J Clin Invest.

[17] Cummins EP, Keogh CE, Crean D, Taylor CT (2016). The role of HIF in immunity and inflammation. Mol Aspects Med.

[18] Devraj G, Beerlage C, Brune B, Kempf VA (2017). Hypoxia and HIF-1 activation in bacterial infections. Microbes Infect.

[19] Werth N, Beerlage C, Rosenberger C, Yazdi AS, Edelmann M, Amr A (2010). Activation of hypoxia inducible factor 1 is a general phenomenon in infections with human pathogens. PLoS ONE.

[20] Schaffer K, Taylor CT (2015). The impact of hypoxia on bacterial infection. FEBS J.

[21] Schaible B, Taylor CT, Schaffer K (2012). Hypoxia increases antibiotic resistance in Pseudomonas aeruginosa through altering the composition of multidrug efflux pumps. Antimicrob Agents Chemother.

[22] Yu C, Zhan X, Liang T, Chen L, Zhang Z, Jiang J (2021). Mechanism of hip arthropathy in Ankylosing Spondylitis: abnormal myeloperoxidase and phagosome. Front Immunol.

[23] Chen Q, Zhou H, Rong W (2022). Circular RNA_0078767 upregulates Kruppel-like factor 9 expression by targeting microRNA-889, thereby inhibiting the progression of osteosarcoma. Bioengineered.

[24] Sun X, Xin S, Jin L, Zhang Y, Ye L (2022). Neurexophilin 4 is a prognostic biomarker correlated with immune infiltration in bladder cancer. Bioengineered.

[25] Kanehisa M, Goto S (2000). KEGG: kyoto encyclopedia of genes and genomes. Nucleic Acids Res.

[26] Kanehisa M (2019). Toward understanding the origin and evolution of cellular organisms. Protein Sci.

[27] Kanehisa M, Furumichi M, Sato Y, Kawashima M, Ishiguro-Watanabe M (2023). KEGG for taxonomy-based analysis of pathways and genomes. Nucleic Acids Res.

[28] Schriml LM, Arze C, Nadendla S, Chang YW, Mazaitis M, Felix V (2012). Disease Ontology: a backbone for disease semantic integration. Nucleic Acids Res.

[29] Jiang J, Zhan X, Qu H, Liang T, Li H, Chen L (2022). Upregulated of ANXA3, SORL1, and neutrophils may be key factors in the Progressionof Ankylosing Spondylitis. Front Immunol.

[30] Wang N, Zhang H, Li D, Jiang C, Zhao H, Teng Y (2021). Identification of novel biomarkers in breast cancer via integrated bioinformatics analysis and experimental validation. Bioengineered.

[31] Du S, Zeng F, Sun H, Liu Y, Han P, Zhang B (2022). Prognostic and therapeutic significance of a novel ferroptosis related signature in colorectal cancer patients. Bioengineered.

[32] Jia W, Liu X, Wang Y, Pedrycz W, Zhou J (2022). Semisupervised learning via axiomatic fuzzy set theory and SVM. IEEE Trans Cybern.

[33] Stoltzfus JC (2011). Logistic regression: a brief primer. Acad Emerg Med.

[34] Rabaglino MB, Salilew-Wondim D, Zolini A, Tesfaye D, Hoelker M, Lonergan P (2023). Machine-learning methods applied to integrated transcriptomic data from bovine blastocysts and elongating conceptuses to identify genes predictive of embryonic competence. FASEB J.

[35] Liu Y, Bhagwate A, Winham SJ, Stephens MT, Harker BW, McDonough SJ (2022). Quality control recommendations for RNASeq using FFPE samples based on pre-sequencing lab metrics and post-sequencing bioinformatics metrics. BMC Med Genomics.

[36] Hu S, Shen C, Yao X, Zou Y, Wang T, Sun X (2022). m6A regulator-mediated methylation modification patterns and immune microenvironment infiltration characterization in osteoarthritis. BMC Med Genomics.

[37] Liu M, Yang J, Wang J, Deng L (2020). Predicting miRNA-disease associations using a hybrid feature representation in the heterogeneous network. BMC Med Genomics.

[38] Wishart DS, Feunang YD, Guo AC, Lo EJ, Marcu A, Grant JR (2018). DrugBank 5.0: a major update to the DrugBank database for 2018. Nucleic Acids Res.

[39] Davis JM, Ramakrishnan L (2009). The role of the granuloma in expansion and dissemination of early tuberculous infection. Cell.

[40] Chao MC, Rubin EJ (2010). Letting sleeping dos lie: does dormancy play a role in tuberculosis?. Annu Rev Microbiol.

[41] Galagan JE, Minch K, Peterson M, Lyubetskaya A, Azizi E, Sweet L (2013). The Mycobacterium tuberculosis regulatory network and hypoxia. Nature.

[42] Yang H, Wang F, Guo X, Liu F, Liu Z, Wu X (2021). Interception of host fatty acid metabolism by mycobacteria under hypoxia to suppress anti-TB immunity. Cell Discov.

[43] Trauner A, Lougheed KE, Bennett MH, Hingley-Wilson SM, Williams HD (2012). The dormancy regulator DosR controls ribosome stability in hypoxic mycobacteria. J Biol Chem.

[44] Sambarey A, Devaprasad A, Baloni P, Mishra M, Mohan A, Tyagi P (2017). Meta-analysis of host response networks identifies a common core in tuberculosis. NPJ Syst Biol Appl.

[45] Cui Z, Hwang SM, Gomes AV (2014). Identification of the immunoproteasome as a novel regulator of skeletal muscle differentiation. Mol Cell Biol.

[46] Dahlmann B (2005). Proteasomes Essays Biochem.

[47] Shoji T, Kikuchi E, Kikuchi J, Takashima Y, Furuta M, Takahashi H (2020). Evaluating the immunoproteasome as a potential therapeutic target in cisplatin-resistant small cell and non-small cell lung cancer. Cancer Chemother Pharmacol.

[48] Ihle JN (2001). The Stat family in cytokine signaling. Curr Opin Cell Biol.

[49] Casanova JL, Holland SM, Notarangelo LD (2012). Inborn errors of human JAKs and STATs. Immunity.

[50] Dupuis S, Dargemont C, Fieschi C, Thomassin N, Rosenzweig S, Harris J (2001). Impairment of mycobacterial but not viral immunity by a germline human STAT1 mutation. Science.

[51] Rincon-Arevalo H, Aue A, Ritter J, Szelinski F, Khadzhynov D, Zickler D (2022). Altered increase in STAT1 expression and phosphorylation in severe COVID-19. Eur J Immunol.

[52] Liang T, Chen J, Xu G, Zhang Z, Xue J, Zeng H (2022). STAT1 and CXCL10 involve in M1 macrophage polarization that may affect osteolysis and bone remodeling in extrapulmonary tuberculosis. Gene.

[53] Garbi N, Tanaka S, van den Broek M, Momburg F, Hammerling GJ (2005). Accessory molecules in the assembly of major histocompatibility complex class I/peptide complexes: how essential are they for CD8(+) T-cell immune responses?. Immunol Rev.

[54] Strehl B, Seifert U, Kruger E, Heink S, Kuckelkorn U, Kloetzel PM (2005). Interferon-gamma, the functional plasticity of the ubiquitin-proteasome system, and MHC class I antigen processing. Immunol Rev.

[55] Harriff MJ, Burgdorf S, Kurts C, Wiertz EJ, Lewinsohn DA, Lewinsohn DM (2013). TAP mediates import of Mycobacterium tuberculosis-derived peptides into phagosomes and facilitates loading onto HLA-I. PLoS ONE.

[56] Zhang M, Wang X, Zhu Y, Chen S, Chen B, Liu Z (2021). Associations of genetic variants at TAP1 and TAP2 with pulmonary tuberculosis risk among the chinese population. Epidemiol Infect.

[57] Motiee M, Zavaran Hosseini A, Soudi S (2022). Evaluating the effects of Cyclosporine A immunosuppression on mycobacterial infection by inhaling of Cyclosporine A administrated BALB/c mice with live Bacillus Calmette Guerin. Tuberculosis (Edinb).

[58] Simmons JD, Stein CM, Seshadri C, Campo M, Alter G, Fortune S (2018). Immunological mechanisms of human resistance to persistent Mycobacterium tuberculosis infection. Nat Rev Immunol.

[59] Prezzemolo T, Guggino G, La Manna MP, Di Liberto D, Dieli F, Caccamo N (2014). Functional signatures of human CD4 and CD8 T cell responses to Mycobacterium tuberculosis. Front Immunol.

[60] Snast I, Bercovici E, Solomon-Cohen E, Avni T, Shitenberg D, Hodak E (2019). Active tuberculosis in patients with psoriasis receiving biologic therapy: a systematic review. Am J Clin Dermatol.

[61] Ge MQ, Ho AW, Tang Y, Wong KH, Chua BY, Gasser S (2012). NK cells regulate CD8 + T cell priming and dendritic cell migration during influenza a infection by IFN-gamma and perforin-dependent mechanisms. J Immunol.

[62] Hewison M (2011). Antibacterial effects of vitamin D. Nat Rev Endocrinol.

[63] Crowle AJ, Ross EJ, May MH (1987). Inhibition by 1,25(OH)2-vitamin D3 of the multiplication of virulent tubercle bacilli in cultured human macrophages. Infect Immun.

[64] Chan J, Flynn J (2004). The immunological aspects of latency in tuberculosis. Clin Immunol.

[65] Banchereau J, Steinman RM (1998). Dendritic cells and the control of immunity. Nature.

[66] Ingulli E, Mondino A, Khoruts A, Jenkins MK (1997). In vivo detection of dendritic cell antigen presentation to CD4(+) T cells. J Exp Med.

[67] Lande R, Giacomini E, Grassi T, Remoli ME, Iona E, Miettinen M (2003). IFN-alpha beta released by Mycobacterium tuberculosis-infected human dendritic cells induces the expression of CXCL10: selective recruitment of NK and activated T cells. J Immunol.

[68] Meermeier EW, Harriff MJ, Karamooz E, Lewinsohn DM (2018). MAIT cells and microbial immunity. Immunol Cell Biol.

[69] De Libero G, Mori L (2014). The T-Cell response to lipid antigens of Mycobacterium tuberculosis. Front Immunol.

[70] Huang S (2016). Targeting Innate-Like T cells in tuberculosis. Front Immunol.

[71] Shen L, Frencher J, Huang D, Wang W, Yang E, Chen CY (2019). Immunization of Vgamma2Vdelta2 T cells programs sustained effector memory responses that control tuberculosis in nonhuman primates. Proc Natl Acad Sci U S A.

