# Supplementary material for: Proteomic analysis to identification of hypoxia related markers in spinal tuberculosis: a study based on weighted gene co-expression network analysis and machine learning
Source: BMC Med Genomics. 2023 Jun 20;16:142. doi: 10.1186/s12920-023-01566-z (PMC10280914; doi:10.1186/s12920-023-01566-z)
Supplement: Supplementary file 3 — Supplementary Material 3 [file 12920_2023_1566_MOESM3_ESM.docx]

The result of quantitative repeatability:


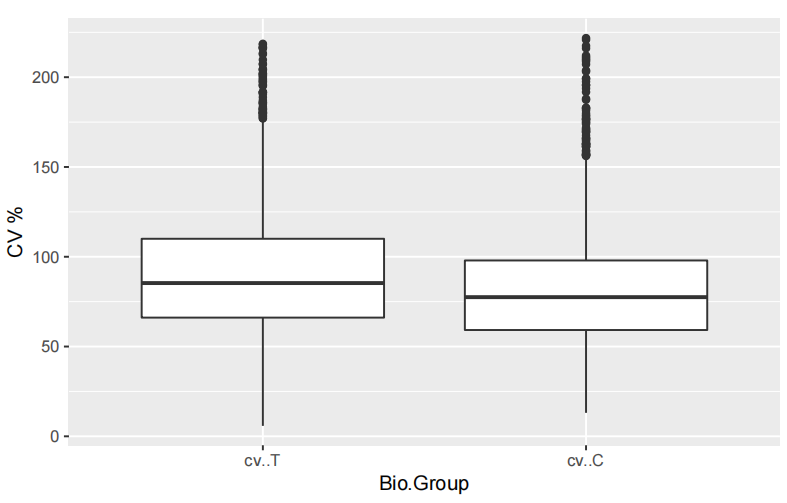


**Figure S1** Distribution of quantitative coefficient of variation between biological repeats. T, spinal tuberculosis; C, control group；CV, coefficient of variation.


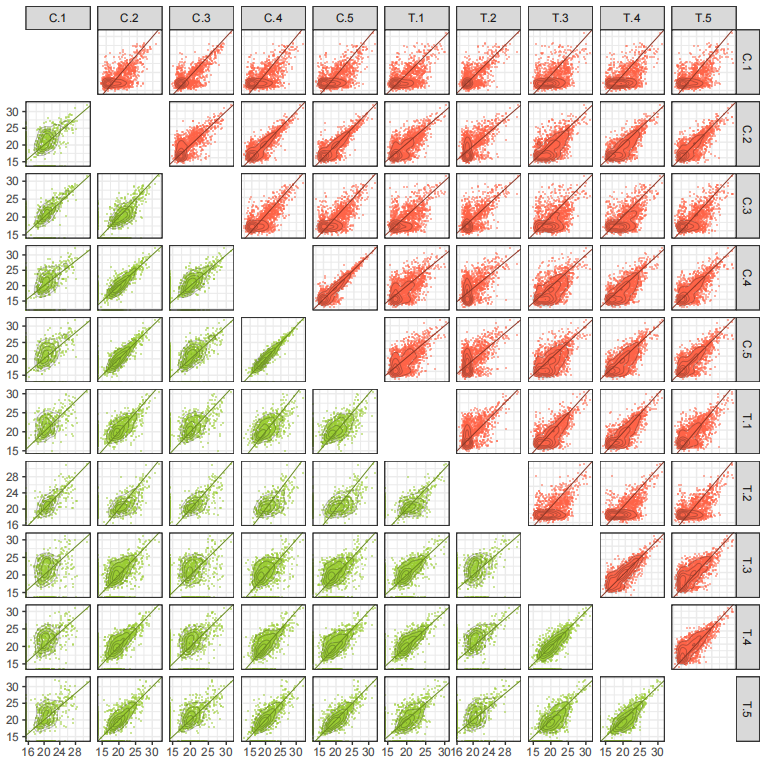


**Figure S2** The quantitative correlation analysis between samples. T, spinal tuberculosis; C, control group.
